# Boosting the Understanding and Approval of Anti‐Corona Measures–Reducing Exponential Growth Bias and its Effects through Educational Nudges

**DOI:** 10.1111/spsr.12479

**Published:** 2021-09-29

**Authors:** Sebastian Jäckle, Felix Ettensperger

**Affiliations:** ^1^ Albert‐Ludwigs‐Universität Freiburg

**Keywords:** exponential growth bias, nudges, COVID‐19, Coronavirus, statistical literacy, political framing

## Abstract

One major problem of compliance with anti‐coronavirus measures originates from the so‐called exponential growth bias, i.e. the cognitive distortion of systematically underestimating exponential growth and its consequences. We replicate an Amazon MTurk experiment regarding the spread of SARS‐CoV‐2 that was conducted in the general US population during the first wave of the pandemic in March 2020 dealing with this bias. Using a least‐likely‐design‐approach, we find a similarly strong bias in our sample of German students in November 2020. Nevertheless, this bias can be reduced by one simple educational intervention. Furthermore, participants who received these educational nudges showed a considerably higher approval rating for contact restrictions. This effect is robust to different analytical techniques and the inclusion of controls. Complementing political statements about the exponential spread of the virus – which often only mention the name of the phenomenon – with simple educational nudges could help the public better understand the need for encroachments on personal liberties.

## Zusammenfassung

Ein Hauptproblem bei der Einhaltung von Anti‐Corona‐Maßnahmen ist der sogenannte *Exponential Growth Bias*, d.h. die systematische Unterschätzung des exponentiellen Wachstums und seiner Folgen. Dieser Artikel repliziert ein Experiment zu diesem Bias, das während der ersten Welle der Sars‐Cov‐2‐Pandemie im März 2020 über Amazon MTurk in den USA durchgeführt wurde. Einer *least‐likely*‐Logik folgend, finden wir in unserer Stichprobe deutscher Studierender im November 2020 einen ähnlich starken Bias. Dieser lässt sich jedoch durch eine einfache pädagogische Intervention bedeutend reduzieren. Teilnehmer, die diesen pädagogischen Anstoß (Nudge) erhalten haben, zeigen ausserdem eine deutlich höhere Zustimmung zu Kontaktbeschränkungen. Dieser Effekt ist robust gegenüber verschiedenen Analysemethoden und der Einbeziehung von Kontrollen. Ein pädagogischer Nudging‐Ansatz, der exponentielles Wachstum nicht nur als Begriff benennt, sondern es der Bevölkerung begreiflich macht, könnte entsprechend dabei helfen, die Gefahren einer exponentiellen Ausbreitung des Virus zu verdeutlichen und die Öffentlichkeit somit auch von der Notwendigkeit von Eingriffen in persönliche Freiheiten zu überzeugen.

## Résumé

La sous‐estimation systématique de la croissance exponentielle et de ses conséquences, que l’on appelle « exponential growth bias », figure parmi les problèmes majeurs dans le respect des mesures anti‐Covid. Cette note de recherche reproduit une expérience au sujet de cette erreur qui a été conduite à l’aide de Amazon MTurk aux États‐Unis pendant la première vague du Covid‐19, en mars 2020. Suivant une logique du « least likely case », nous trouvons une sous‐estimation similaire dans notre échantillon d’étudiants allemands en novembre 2020. Or, cette erreur peut être réduite par le biais d’une simple intervention pédagogique. De plus, les participants ayant reçu une incitation pédagogique (« nudge ») montrent une plus forte adhésion à la réduction des contacts. Cet effet résiste à l’application de différentes méthodes d’analyse et aux contrôles. Une approche de « nudging » pédagogique, qui irait au‐delà d’une simple mention de la croissance exponentielle, pourrait donc contribuer à mieux démontrer les dangers de la propagation exponentielle du virus et à convaincre la population de la nécessité d’atteintes à la liberté individuelle.

## INTRODUCTION

During the COVID‐19 pandemic, several non‐pharmaceutical interventions, including social distancing measures, were enacted by political actors at various levels of the German federal system. It became apparent that the effect of these measures depends on the approval as well as compliance of the population. Since these measures represent encroachments on personal liberties, they are generally met with skepticism in a liberal democratic society. In addition to coercive state measures – which in a constitutional state must always satisfy the requirement of proportionality – compliance with the rules can be especially ensured if the population is generally supportive of them and considers the rules to be correct and appropriate. This is the starting point for the present research note. Its main argument is that the so‐called exponential growth bias (EGB), to which humans are generally subject, means that we often fail to recognize the seriousness of developments during the COVID‐19 pandemic, or recognize them too late. However, we will show that it is possible to mitigate this bias through an educational nudging approach and hence to ultimately increase consent to social distancing measures.

## RELEVANCE

Compared to most European countries, Germany was only mildly affected by the first wave of the COVID‐19 pandemic in spring 2020. This fact and the very low incidence numbers during summer potentially made it more difficult for politicians to persuade citizens of the necessity of social distancing measures before and at beginning of the second wave which accelerated considerably in Germany in October 2020. Surveys showed that while the then existing anti‐Corona measures were largely accepted, they were also seen as sufficient to prevent the virus from spreading exponentially (Betsch, 2020). Furthermore, a rising polarization between societal groups with regard to their stance towards the pandemic and the government measures were detected (Wagschal et al., 2020). These peculiarities make it interesting to analyze Germany. At the same time, governments of many other countries struggled similarly to find acceptance for non‐pharmaceutical anti‐Corona measures that restrict personal freedoms. The results of this research note may thus also be relevant for countries other than Germany. Since exponential growth is considered to be one of the major underlying elements of the COVID‐19 pandemic (as of many other pandemics before), a good understanding of this type of growth in the general public could help raise awareness and consequently also the approval of government measures that restrict individual rights. Politicians used the term “exponential growth” often during the pandemic. It is unclear, however, whether the mere repetition of this term is sufficient to make the population aware of the seriousness of the situation. It therefore makes sense to test to what extent the general public is able to understand the consequences of exponential growth and whether a better understanding also impacts their attitude towards anti‐Corona measures. In more general terms, this research could be an example of how social and behavioral science can help support COVID‐19 pandemic responses (Bavel et al., 2020).

## EXPONENTIAL GROWTH BIAS AND EDUCATIONAL NUDGES

Exponential growth is anything but rare in many fields and disciplines. We see it, for example, in biology when microorganisms grow in a petri dish; in physics, when describing the nuclear chain reaction; in economics when it comes to economic growth^1^; in computer science, when describing the growth of processing power via Moore’s law; as well as in social media when it comes to the spread of viral content. Yet, for most people exponential growth is difficult to understand and, most importantly, we are not good at fully realizing its consequences. This is by no means a new finding. The ancient Indian tale of the rice grain on the chessboard already showed which enormous masses (in this case of rice) can arise when exponential growth is present; it also showed how much this form of growth eludes common sense. Yet, the way this legend is told, as well as similar educational examples of exponential growth (e.g. the question of how many times you can fold a piece of paper in half), tend to be perceived as a mathematical curiosity with no actual equivalent in everyday life. Studies that demonstrate the real‐world implications of EGB have falsified this assumption by showing the effects of EGB with regard to financial decisions on (retirement) savings and debts (Foltice & Langer, 2018; Goda et al., 2019) or household consumption plans (Levy & Tasoff, 2016; Stango & Zinman, 2009) and find EGB to be linked to a person’s general financial literacy (Almenberg & Gerdes, 2012). In addition, people tend to overestimate their own ability to predict exponential growth (Cordes et al., 2019; Levy & Tasoff, 2017) and to perceive their problems in that regard as manifestations of poor mental calculation skills instead of manifestations of a deeper, underlying problem: the poor conceptual understanding of exponential growth. Although, according to these studies, EGB seems to be a universal problem among all people, there is evidence that the strength of the bias may well depend on cultural context (Keren, 1983). With respect to COVID‐19, online experiments have proven EGB to exist and to have implications for the perception of safety regulations (e.g. social distancing measures), future economic prospects and the riskiness of people’s investment strategies (Banerjee et al., 2021; Banerjee & Majumdar, 2020; Lammers et al., 2020).

However, studies on EGB also show that targeted educational measures can improve these biased estimations as well as their implications (Foltice & Langer, 2017; Goda et al., 2014). One simple option is the use of nudges in communication strategies. Educational research demonstrates that particularly transparent type 2 nudges, i.e. nudges that also trigger reflective thinking and aim at changing deliberate choices, could be adjuvant with regard to EGB (Weijers et al., 2020). Schonger and Sele furthermore demonstrate that regarding infectious diseases, the way exponential growth is communicated, i.e. framed, matters for a person’s evaluation of the situation and the “assessment of the benefits of non‐pharmaceutical interventions” (Schonger & Sele, 2020: 1).

## THE EXPERIMENT

Our experiment adapts parts of the analysis by Lammers et al. (2020), which we describe here in short, before explaining the experimental setup of our study in more detail.

During the first wave, in mid‐March 2020, Lammers and colleagues conducted three online experiments about EGB in the USA via Amazon MTurk. Participants from the general public were given information on the number of infected persons and the doubling time. In three distinct studies, they were then asked to estimate how this number had changed in the days before, and to predict how it would develop in the following 15 days. In the first study, the authors find substantial evidence for EGB when it comes to the spread of SARS‐Cov‐2. Participants “erroneously perceive the virus’s exponential growth in largely linear terms” (Lammers et al., 2020: 16266). In the two other sub‐studies, it was demonstrated not only that comparatively simple educational measures were suitable for minimizing the EGB, but also that participants who received this additional help showed greater agreement with social distancing measures as compared to people in the control group. The support intervention in their study was provided in the form of additional arithmetical steps, allowing for an easier step‐by‐step estimation of the final number of infected persons.

We replicated this experiment in a situation that could be described as less likely case for EGB impact: We invited students from introductory political science methods courses to participate in a survey on the overall impact of the coronavirus pandemic on students’ lives in order to generate a dataset that could then be used in class for teaching – no further incentives were given. The students were unaware of the fact that it also comprised the exponential growth experiment.^2^ The overall educational background of this group of university students can plausibly be regarded as higher than in a sample from the general (US) public. Most students were currently in their first semester, a smaller proportion in their third or a higher semester. Since most of these participants had taken obligatory mathematics classes in high school until very recently, they should be able to remember the mathematical concept of exponential growth better and fall back on it more easily than a randomly selected group of people. Additionally, by the time of the experiment at the end of November 2020, it was very likely that participants had frequently been confronted with the concepts of exponential growth, doubling times and the R‐value in the media in relation to SARS‐Cov‐2. These concepts had been mentioned by politicians and news reports repeatedly in the previous months; it is thus reasonable to assume that these students were more exposed to these concepts as compared to the participants in the study of Lammers et al. from the first wave in March 2020. For these reasons, we expect it to be less likely to find a similarly strong EGB as Lammers et al.; finding one, however, would have particularly high significance.

In our study, participants were randomly assigned to either a control or a treatment condition. Both groups received the following information:

As of March 22, there were about 25,000 confirmed infections in the United States, according to Johns Hopkins University. Three days later, on March 25, there were about 50,000.

**FIGURE 1:**
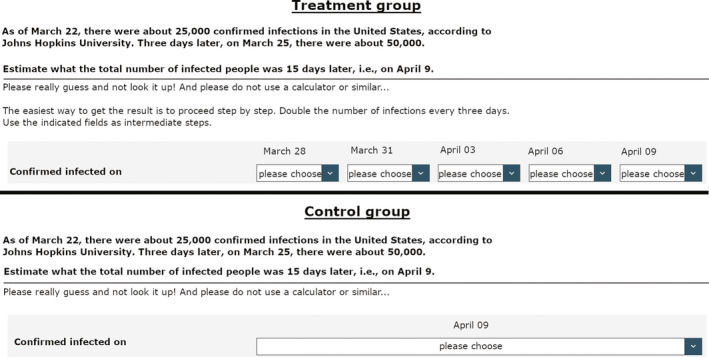
Treatment and control conditions of the experiment (translated from German) [Colour figure can be viewed at wileyonlinelibrary.com]

None of the groups received explicit information about the fact that the virus spreads exponentially, but at the time of the experiment in November 2020, it can be assumed for Germany that this is largely common knowledge and that everyone has heard about it – which admittedly does not mean that everyone has understood what exponential growth means. Both groups were asked to estimate the total number of infected persons 15 days later, on April 9^th^, without using a calculator or other mathematical tools (e.g. written calculation). The control group had to complete this task without any further help. The treatment group, however, received the information that the easiest way to estimate this number was to double the number of infected persons every three days. Participants in the treatment group also had the possibility to write down the number of expected cases after 3, 6, 9 and 12 days before they had to give their estimate for the number of infected persons on April 9. Figure 1 gives an overview of the treatment and control conditions translated to English. For the original German wording see Online Appendix, Figure A1.

Participants in the treatment group thus received educational nudges but still had to do the estimation on their own in a simple step‐by‐step calculation process, while participants in the control group had to understand the mathematical task on their own and had to estimate the final value of all five iterations of case doubling during the entire timeframe within one single step. After this estimation, we asked participants about their approval of four tangible anti‐Corona measures that had been discussed or implemented at that time of the survey. Specifically, participants had to rate to what extent they approved of contact restrictions in general, the closing of restaurants, the mandatory use of face masks and hybrid teaching at schools. The approval ratings were recorded on a 10‐point Likert‐scale (1: strongly oppose; 10: strongly support).

A total of 128 participants provided information in the online questionnaire, 61 were in the treatment group, 63 in the control group. Four cases had to be excluded because participants did not provide either an answer to the estimation question or the approval ratings regarding anti‐Corona measures.

The overall research design of our study resembles the design in study 3 of Lammers et al. Our analysis can thus, on the one hand, be regarded as a replication study; on the other hand, compared to Lammers et al., our study includes some enhancements: First, as mentioned above, our student sample and the timing of the experiment make it less likely for participants to be subject to an EGB – if we find one, however, this means that this sort of bias is certainly widespread in the population. Second, we analyze the four approval ratings for anti‐Corona measures independently while Lammers et al. used the mean rating of five single measures. Third, we also included a more diverse set of control variables including social trust, political left‐right self‐evaluation, and perceived personal burden due to COVID‐19, which were all retrieved during the survey before the actual experiment. This allows us to evaluate not only if simple educational nudges help participants to better grasp the consequences of an exponentially spreading virus, but also if this stimulus eventually impacts the perception of specific anti‐Corona measures. Using information on further variables, we are able to compare the strength of the educational nudges effect to the effect of other potentially relevant factors.

## ANALYSIS

Our analysis proceeds in three steps: first, we compare the estimations made by the control group and the treatment group in our setup with the results from Lammers et al. The second step examines whether the treatment group has higher approval rates on four anti‐Corona measures queried after the experimental task than the control group. In a third step, we control for other factors that could explain a difference in the approval rates in a regression model in order to safeguard a possible educational nudging effect as much as possible (see Jäckle & Ettensperger, 2021, for replication data).

**FIGURE 2:**
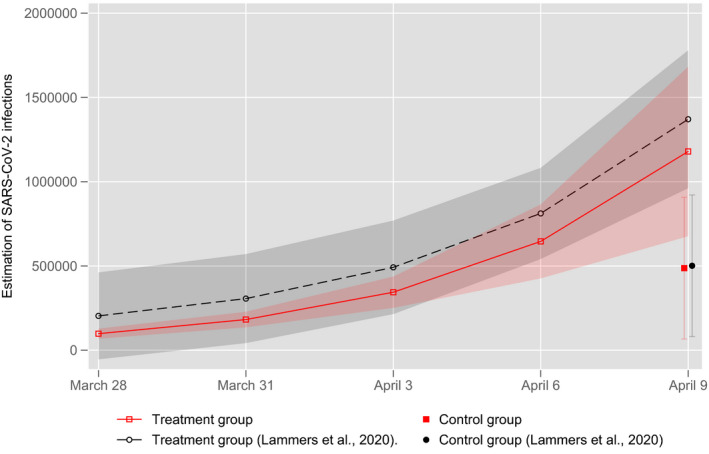
Estimated number of infections with SARS‐Cov‐2 made by the participants (Mean ± 1 SD) [Colour figure can be viewed at wileyonlinelibrary.com]

Lammers et al. found that subjects who received the educational nudges estimated the number of infections on average 173% higher than subjects in the control group (2020: 16266). Figure 2 shows that although the difference in the estimations between the two groups in our study is slightly lower, the treatment group still produces 142% higher mean‐estimates than the control group for April 9. Interestingly, the estimations of the intermediate steps in the treatment group are consistently lower in our sample than those reported by Lammers et al. However, this does not necessarily mean that educational nudges worked worse in our student sample than in the reproduced study. Rather, the standard deviations, which remain constant over the entire range in Lammers et al., show that in their MTurk sample, a large number of participants apparently provided very high, unrealistic estimates already at the first intermediate arithmetic steps. In comparison, the variance of the estimates in our sample increases steadily as one would expect. One plausible explanation for this deviation is that a significant number of the respondents (10‐15%) in the Lammers et al. study did not adequately read the questions and/or clicked the answers randomly.^3^ In most studies this might not be a big problem, but in this specific study it is: this random response pattern does not generate random white noise, but a systematic bias towards higher estimations which subsequently may distort the regression analyses based on it. While this point is clearly not the main finding of our paper, we believe it to be an important one – particularly since the use of Amazon Mturk is growing in social sciences in general. While Mturk clearly has many pros (particularly in terms of data accessibility), our re‐analysis of the Lammers et al. data reinforces existing criticisms of the validity of this data source for survey research (Chmielewski & Kucker, 2020; Kennedy et al., 2020).

Notwithstanding these methodological issues, the results of our experiment confirm the main finding of Lammers et al. (2020): there is a clear EGB in the estimation of the infection incidence of the COVID‐19 pandemic. However, this bias can be reduced significantly through educational nudges. All in all, the bias and the nudging effect we find are comparable in magnitude to those found by Lammers et al.

**FIGURE 3:**
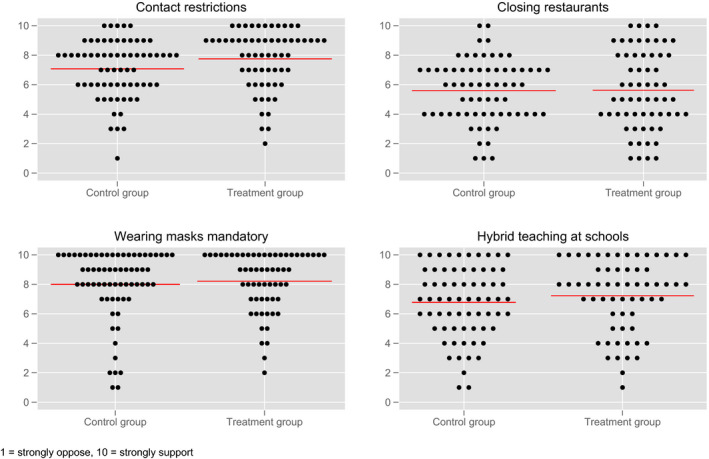
Approval ratings in treatment and control group (+ group means) [Colour figure can be viewed at wileyonlinelibrary.com]

Lammers et al. show that subjects in the experimental condition “were significantly more supportive of measures to increase social distancing and a lockdown than control participants (P = 0.024)” (2020: 12622). To test this effect, we asked participants about their approval of these measures after they had completed the estimation task. We asked to what extent they approved of the following four anti‐COVID‐19 measures: contact restrictions (e.g. no more than 2 households are allowed to meet), closing of restaurants, mandatory use of face masks in the public and hybrid teaching at schools. Figure 3 depicts the distribution of responses.

For the contact restrictions, our experiment supports the finding presented by Lammers et al. Irrespectively of whether we treat the 10‐point approval rating as an ordinal or an interval scale, participants who received the educational nudges were significantly more supportive of contact restrictions than participants in the control group (t‐test: *P* = 0.0367; Mann‐Whitney U‐test: *P* = 0.0134). For the other three approval rating variables, the differences between the control and the treatment group are weaker (hybrid teaching) or virtually non‐existent (closing restaurants and mandatory masks).^4^

**FIGURE 4:**
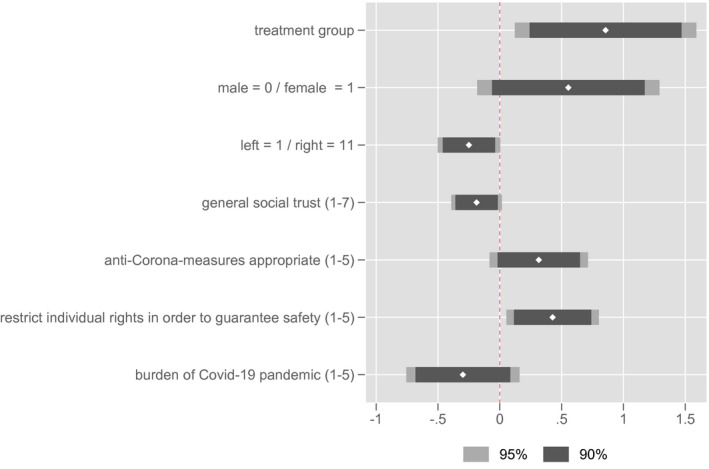
Approval of contact restrictions – OLS‐regression [Colour figure can be viewed at wileyonlinelibrary.com]

Although participants were randomly assigned to the control and treatment group, due to the comparatively small sample size it is still possible that certain differences between the groups exist. These differences could also impact the approval of social distancing measures. To control for possible group differences that may have existed before the educational nudges were given, we had participants answer questions about themselves, their perceptions of social interaction, their attitudes toward the anti‐Corona measures, and their own affectedness by the pandemic before the actual experiment (see Online Appendix, Table A1). Although t‐tests/Mann‐Whitney U‐tests show no differences (not presented), it still makes sense to control for a potential impact of these variables on the approval of contact restrictions, which have been shown to differ substantially between the control group and the treatment group above, via a regression framework. Figure 4 depicts the b‐coefficients and confidence intervals of this OLS‐model in which all Likert‐scaled variables are treated as continuous.

The regression model shows that the control variables indeed impact the approval of contact restrictions. All other things being equal, female participants, persons with a more left political ideology and those with a lower general social trust in other people exhibit a higher approval for contact restrictions. Similarly, participants who believe that the government should restrict individual rights in order to safeguard the social welfare and public security agree more strongly with contact restrictions. At least by trend, this is also the case for participants who, all in all, deem the anti‐Corona measures of the government to be appropriate and those who feel less burdened by the COVID‐19 pandemic and the measures to counter it. All of these effects are plausible with respect to existing data on the approval of anti‐Corona measures in Germany, as for example published by COSMO – COVID‐19 Snapshot Monitoring^5^ or in other recent studies on the subject (Capraro & Barcelo, 2020; Gollwitzer et al., 2020).

Most importantly, however, the effect of the educational nudges in the treatment group remains relevant. According to the standardized beta‐coefficient of 0.212, it is even the strongest of all variables in the model.^6^ The results are also robust to accounting for heteroscedasticity^7^, to treating the Likert‐scale independent variables as categorical, to using an ordinal logit specification and to the test of further variables that account for whether the participants were tested positive themselves or had been in quarantine before (see Online Appendix, Table A2). The adjusted R^2^ of the model is 0.154. However, since this analysis follows an x‐centered research design, a lower R^2^ is justifiable.

The effects of the educational nudges on the approval of restaurant closings, mandatory masks and hybrid teaching at schools are by trend all positive as expected. Yet, none of these effects is significant at the 0.1‐level of significance which could also be a result of the relatively small sample size (see Online Appendix, Figures A4‐A6). Using the estimation of infected persons as an explanatory variable instead of the group membership also yields the expected effect: those participants who give the highest estimates for the number of infections after 15 days (and are thus closest at the exponential growth) approve contact restrictions the most (see Online Appendix, Figure A7).

## DISCUSSION

This research note has shown that an EGB that leads to an erroneous perception of the spread of the coronavirus exists even in a least‐likely situation. The study we replicated (Lammers et al., 2020) used an MTurk sample of US‐Americans and conducted the exponential growth experiment in March 2020 during the first wave of the pandemic. In contrast, our experiment was conducted in Germany in November 2020 and is based on a sample of first semester students. It is thus plausible to assume that the participants in our experiment have a higher level of education than average citizens or the respondents in Lammers et al., even if MTurk users may be more educated than average Americans (Berinsky et al., 2012). Since most of our participants just finished school, the mathematical relationships behind exponential functions should be much more present for them than for other people. Moreover, politicians, government agencies such as the Robert Koch Institute and the media had repeatedly warned against the danger of exponential growth in the number of infections since March. The concept was thus ever‐present, and anyone consuming a minimum of media necessarily had to come into contact with it. For these reasons, we expected an EGB to be less detectable in our experiment than in Lammers et al. (2020). However, our participants were found to be similarly unable to predict the number of infected individuals as the participants in the replicated study.

Nevertheless, our experiment confirms the result of Lammers et al.: giving educational nudges in the form of little helping instructions can significantly enhance the ability of participants to correctly predict exponential growth. From a political science perspective, the most important finding is, however, that such a better understanding of the spread of the pandemic can help raise the approval of social distancing measures. We found that participants who received educational nudges showed a higher approval of contact restrictions after the experiment as compared to those in the control group. For three other measures the differences between the two groups are not as strong or even non‐detectable. This makes sense, since the restriction of contacts is probably the measure that everyone understands to have a major effect on the spread of the virus while the others are less evident (closing restaurants, hybrid teaching at schools) or are already widely accepted by the public (mandatory masks). Our study also shows that the effect of the educational nudges on the approval of contact restrictions remains significant when controlling for other explanatory factors (e.g. gender, political orientation, general acceptance of the anti‐Corona‐measures and the idea that the state should restrict individual rights in order to guarantee safety for the society). The educational nudges even turn out to be the most influential explanatory variable in our regression models: receiving them increases approval of contact restrictions on average by about .8 points on a 10‐point scale.

Every experimental study has limitations. The first limitation of our study is that we did not formally test the least‐likely‐design assumptions. Yet, with a sample consisting only of future academics that just finished high school, it is plausible to assume that this sample is less prone to EGB than a general MTurk‐sample. The same is true for our assumption that in November 2020, the month in which we conducted the experiment, almost all respondents had already heard about the fact that COVID‐19 spreads exponentially. Again, we could not formally test the deviations of our sample in that regard to the sample of Lammers et al. Moreover, the relatively small number of respondents can also be seen as a limitation. Nevertheless, this makes the significant effects we found all the more remarkable. Furthermore, the least‐likely design implies that the results are not representative for the general public. Yet, knowing that the EGB as well as its consequences for the approval of social distancing measures exist even in a situation and with a sample in which this effect is less likely, makes our finding even more relevant. Another limitation concerns the treatment we gave. One could argue that not the educational nudging itself but the implicit hint through this nudging that the underlying process is exponential helped the respondents to better estimate the infection numbers. Yet, given that in November 2020 probably all respondents had heard that COVID‐19 spreads exponentially, we can plausibly assume that the effects we found are solely originating from the educational nudging. Nevertheless, future studies should disentangle these two potential explanations.

The implications of our findings are twofold. First, since in our experiment even persons coming straight out of school exhibit a strong EGB, in the medium to long run the school curricula should be modified accordingly. Exponential growth should be taught less from a purely mathematical point of view but in a way that shows that it as a relevant element of life on earth. This could lead to an increased literacy of such growth processes in general. Second, our results show that the way politicians and the media framed the pandemic, constantly using terms as “the danger of exponential growth” or the necessity to “break the exponential curve” does not mean that the broad public automatically understands what exponential growth actually means for the spread of the pandemic. With respect to our results, communicating the danger of an exponential spread of the virus, politicians and the media should not build on the assumption that the general public understands what exponential growth means. Simply repeating this term over and over again, as politicians have done in the course of the pandemic so far, hardly seems helpful.^8^ Instead, each time exponential growth is mentioned, it should be accompanied by a simple example, e.g. in the form of a sequence of the predicted number of infected persons after one/two/three/four weeks, demonstrating to what extent this growth is different to linear growth. For this purpose, it is also necessary to explain to the public that a model‐based prediction is something fundamentally different than just crystal‐ball gazing. The debates in Germany about the COVID‐19 pandemic unfortunately were often framed in a different way. Model‐based estimations presented by scientists were regarded as panic mongering and the uncertainty of scientific predictions presented by scientists was misinterpreted as mere guessing the future development of the pandemic.

An educational nudging approach could help mitigate one of the major problems of politics today, i.e. the difficulty to persuade people of the necessity of implementing strict measures at a time when the number of infections seems to be extremely low and not hazardous. This is the vicious aspect of exponential growth curves: they start very slowly and lull us into a sense of security, only to rise all the more steeply. Only if politicians succeed in making citizens aware of this fact in a simple way, will the public start to perceive the risk of the spread of COVID‐19 more realistically and alter its social distancing behavior without the need for official restrictions and prohibitions. As this research note has shown, simple educational nudges could be an inexpensive but effective way to go.

### OPEN RESEARCH BADGES

This article has earned an Open Data and Open Materials badges for making publicly available the digitally‐shareable data necessary to reproduce the reported results. The data is available at https://doi.org/10.7910/DVN/TTXZF9.

## Supporting information

Supplementary Material

## Data Availability

Replication data and Stata‐Do‐Files available at Harvard Dataverse. DOI: https://doi.org/10.7910/DVN/TTXZF9

## Footnotes

^1^
Speaking of a constant growth rate as it is common in economics is essentially another way of describing exponential growth.

^2^
The research design and its purpose were later revealed to all participants.

^3^
Taking a closer look at the Lammers et al. replication dataset indeed shows a number of subjects whose estimations cannot easily be explained in a rational manner (e.g., a subject received the following help “If yesterday’s number (50k) doubles by Saturday, then Saturday there are …” and he/she answered 1.6 million. The same subject then dropped his/her estimation at day 9 to 350,000). There is a relatively high number of such apparently contorted answers in their study: e.g. 37 out of 330 give an estimate of 500,000 or higher for the very first step of the calculation (i.e. doubling the number of 50.000 infections). This of course biases the reported means significantly and makes the fact that that Lammers et al. do not indicate the variation of the data in their graphs even more problematic.

^4^
Hybrid teaching (t‐test: *P* = 0.3163; Mann‐Whitney U‐Test: *P* = 0.2446); closing restaurants (t‐test: *P* = 0.9489; Mann‐Whitney U‐Test: *P* = 0.9337; mandatory masks (t‐test: *P* = 0.5150; Mann‐Whitney U‐Test: *P* = 0.7112).

^5^
See the interactive explorer at https://projekte.uni‐erfurt.de/cosmo2020/web and Betsch et al. (2020).

^6^
Additional interaction analyses between treatment and gender as well as ideological position show that by trend, yet not significantly, the educational nudges work better for female respondents and those with a more right‐wing political orientation (see Online Appendix, Figures A2‐A3).

^7^
The Breusch‐Pagan‐test shows no signs of heteroscedasticity, but the White‐test does. Multicollinearity is no issue (max VIF = 1.11).

^8^

Online Appendix Table A3 presents the results of a systematic search in the newspaper *Süddeutsche Zeitung* on politicians’ statements on the corona pandemic containing the term “exponential”. What is obvious is that all but a single statement do not include any sort of explanation as recommended in this study. Only chancellor Merkel used such an educational nudging approach when, at a press briefing at the end of September, she explained how she arrived at a prediction of more than 19,000 daily infections for Christmas. She was indirectly cited in the following way “She had taken the infection events of the past three months, when the numbers had doubled in each case from about 300 to 600, then to 1,200 and finally to 2,400. If one extrapolates the same exponential growth for another three months, one arrives via 4,800 and 9,600 finally at 19,200 by Christmas.” (Fried, 2020, own translation).
