# Supplementary material for: Boosting the Understanding and Approval of Anti‐Corona Measures–Reducing Exponential Growth Bias and its Effects through Educational Nudges
Source: Schweiz Z Polit. 2021 Sep 29;27(4):809–21. doi: 10.1111/spsr.12479 (PMC8662229; doi:10.1111/spsr.12479)
Supplement: Supplementary file 1 — Supplementary Material [file SPSR-27-809-s001.docx]

Boosting the understanding and approval of anti-Corona measures – reducing exponential growth bias and its effects by educational nudges

Sebastian Jäckle, Felix Ettensperger

Online Appendix

Article published in Swiss Political Science Review

# Figure A1: Online experiment (exact wording in German)

Treatment group:


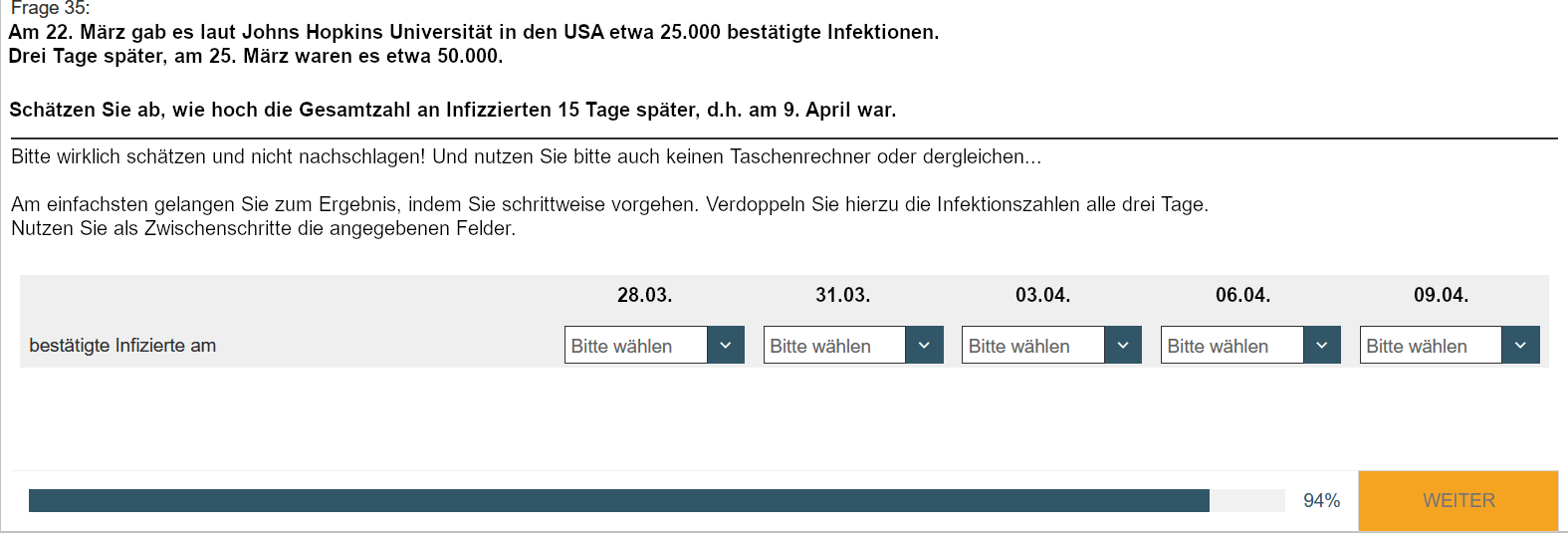


Control group:


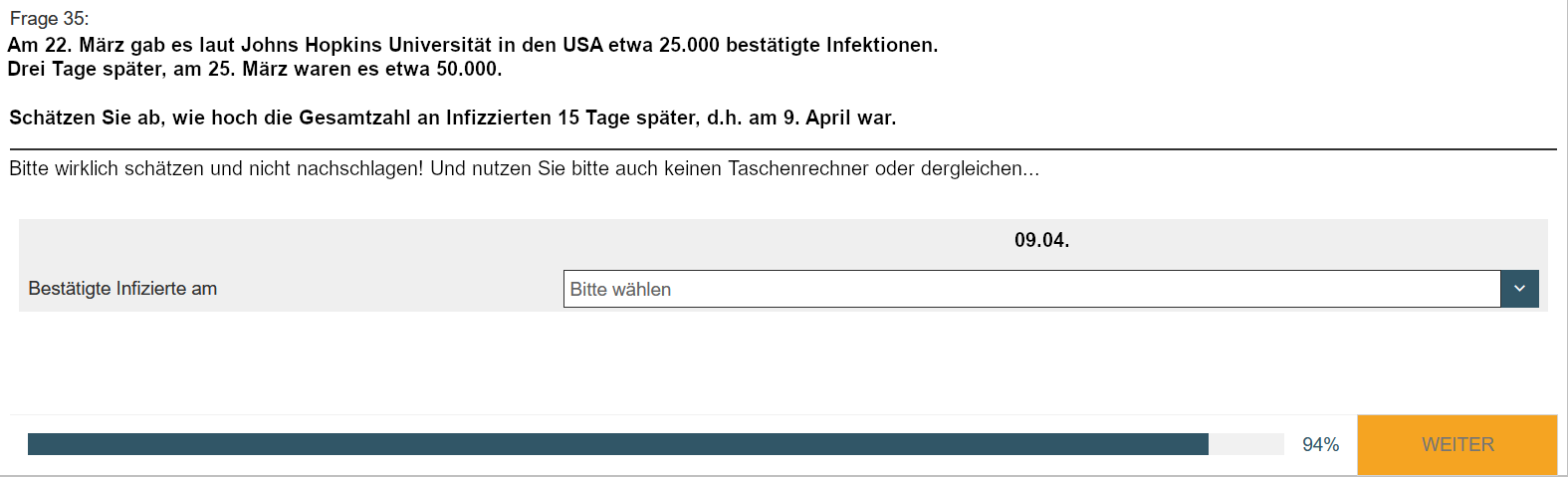


# Figure A2: Interaction between treatment and gender

# Figure A3: Interaction between treatment and political orientation Average marginal effect of treatment (compared to control group) by left-right position (+ 95% CI)

# Figure A4: Approval of closing restaurants – OLS-regression (b-coefficients + CI)


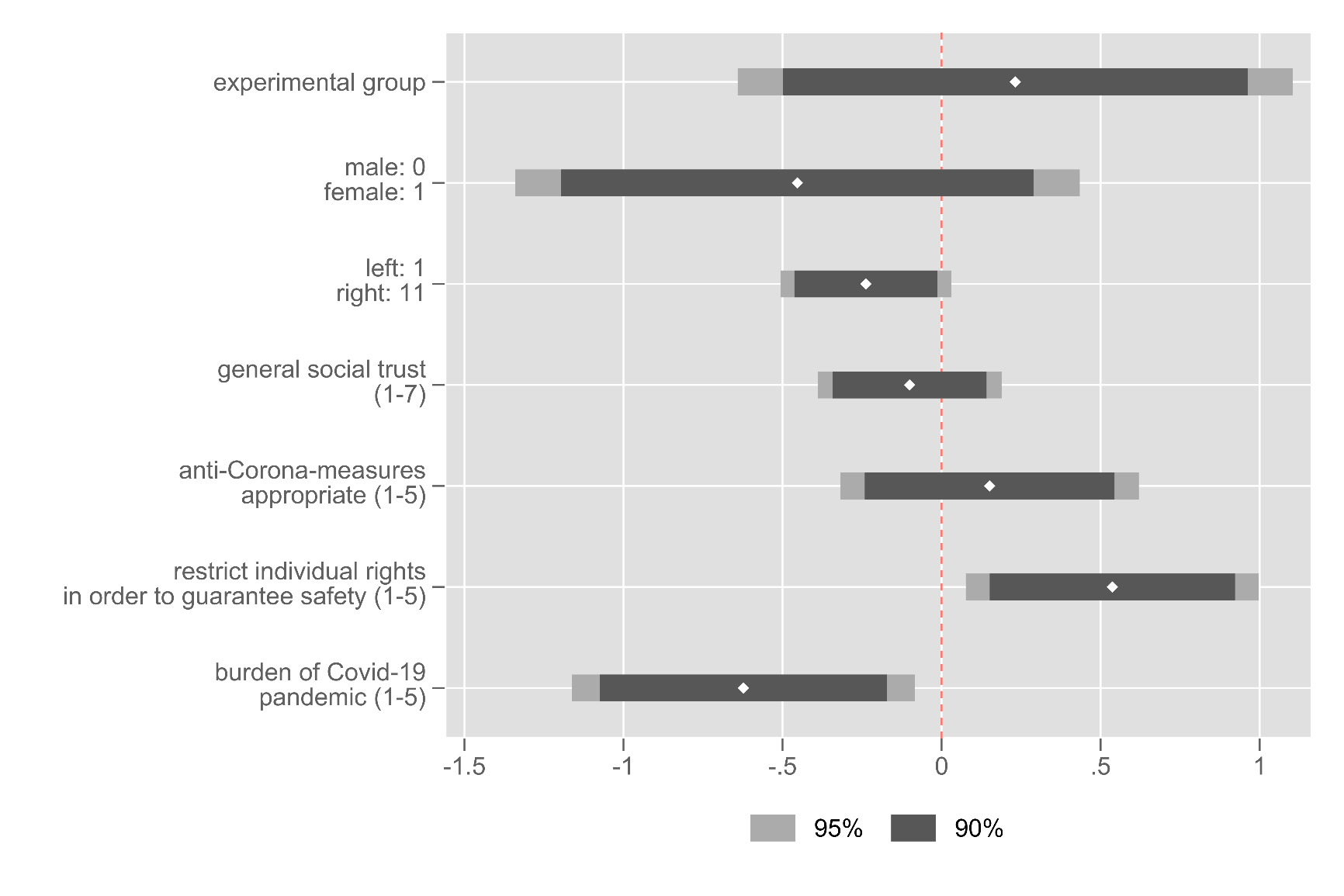


# Figure A5: Approval of mandatory wearing face masks – OLS-regression (b-coefficients + CI)


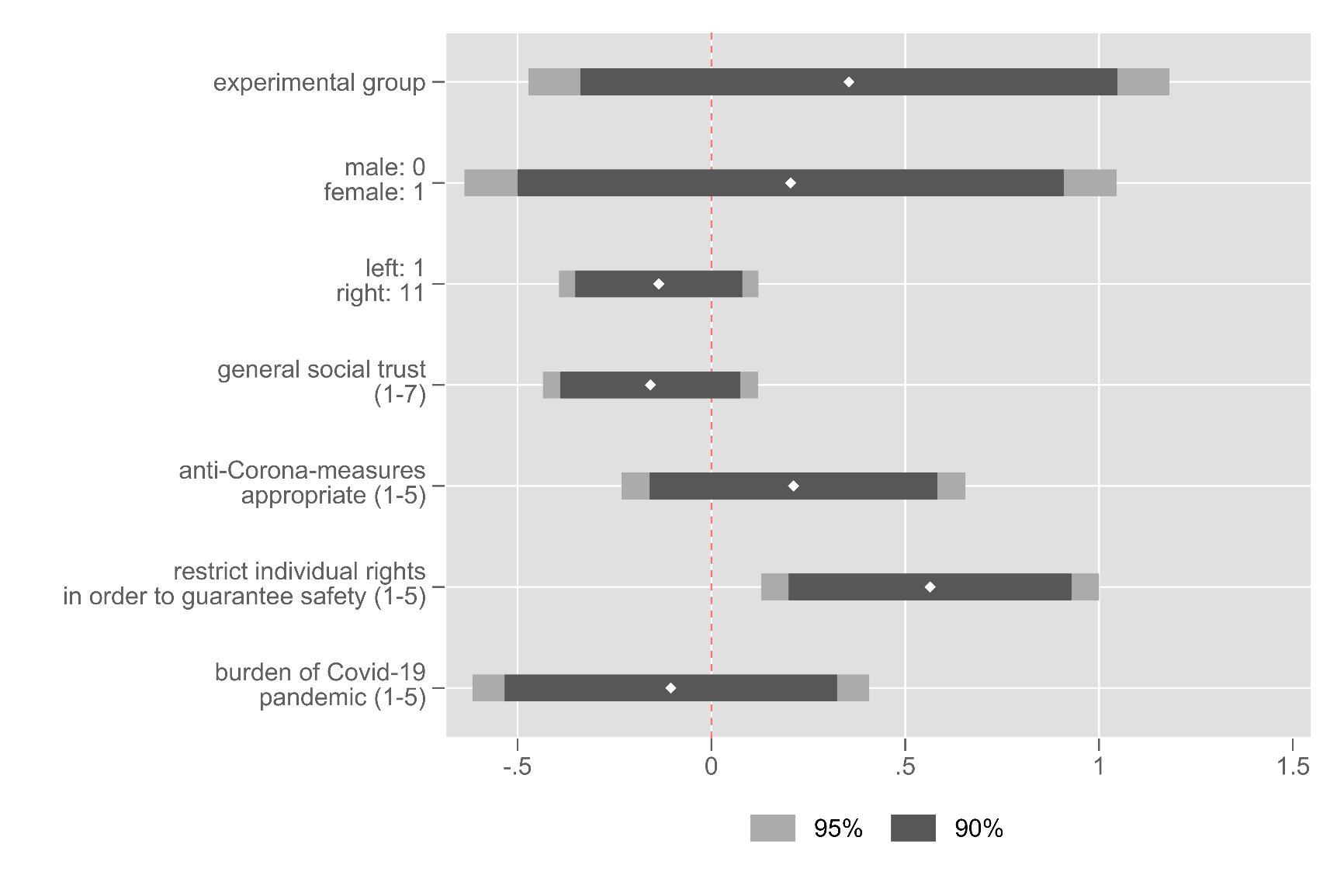


# Figure A6: Approval of hybrid teaching at schools – OLS-regression (b-coefficients + CI)


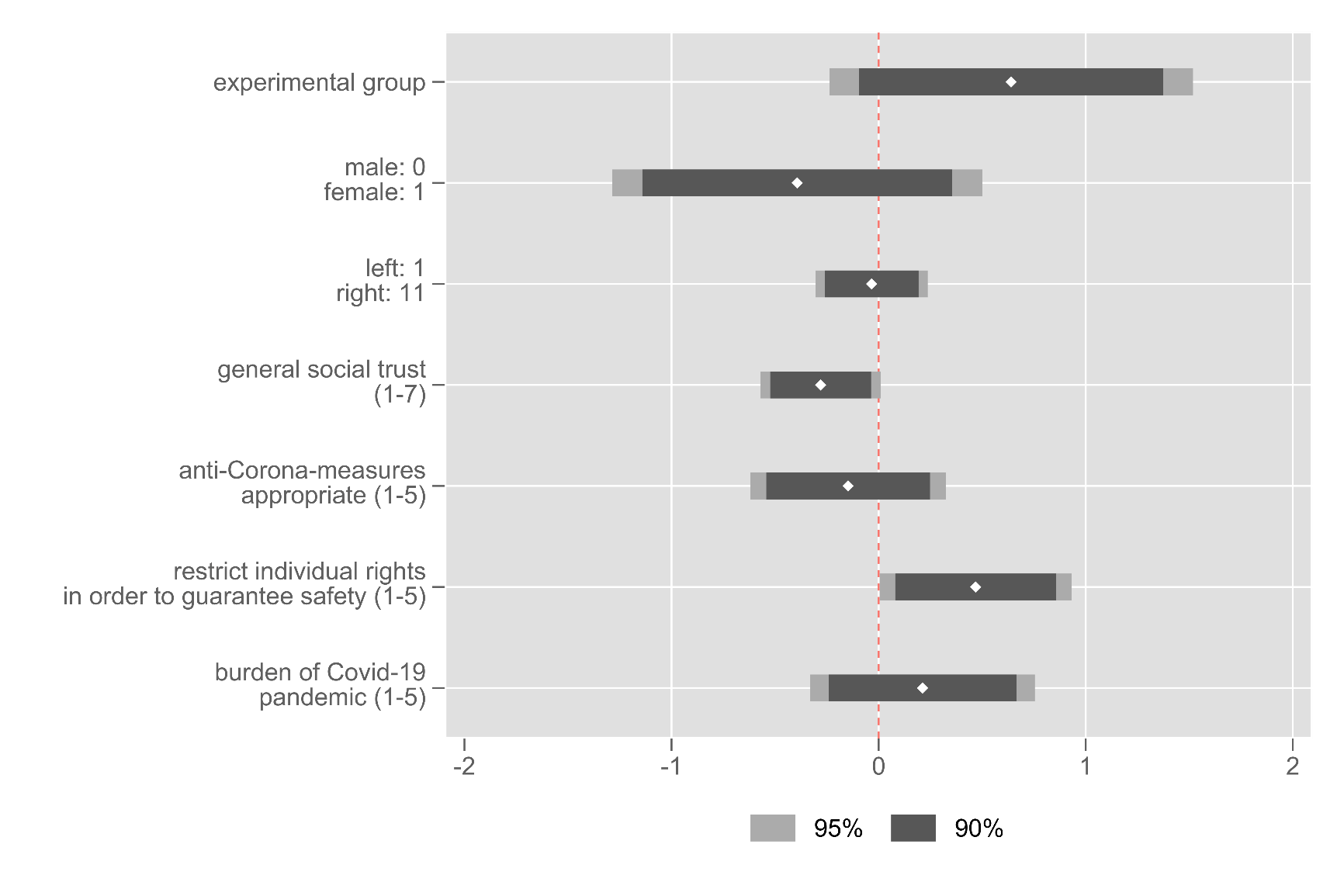


# Figure A7: Approval of closing restaurants – OLS-regression with estimated number of infected persons as explanatory variable instead of experimental/control group (b-coefficients + CI)


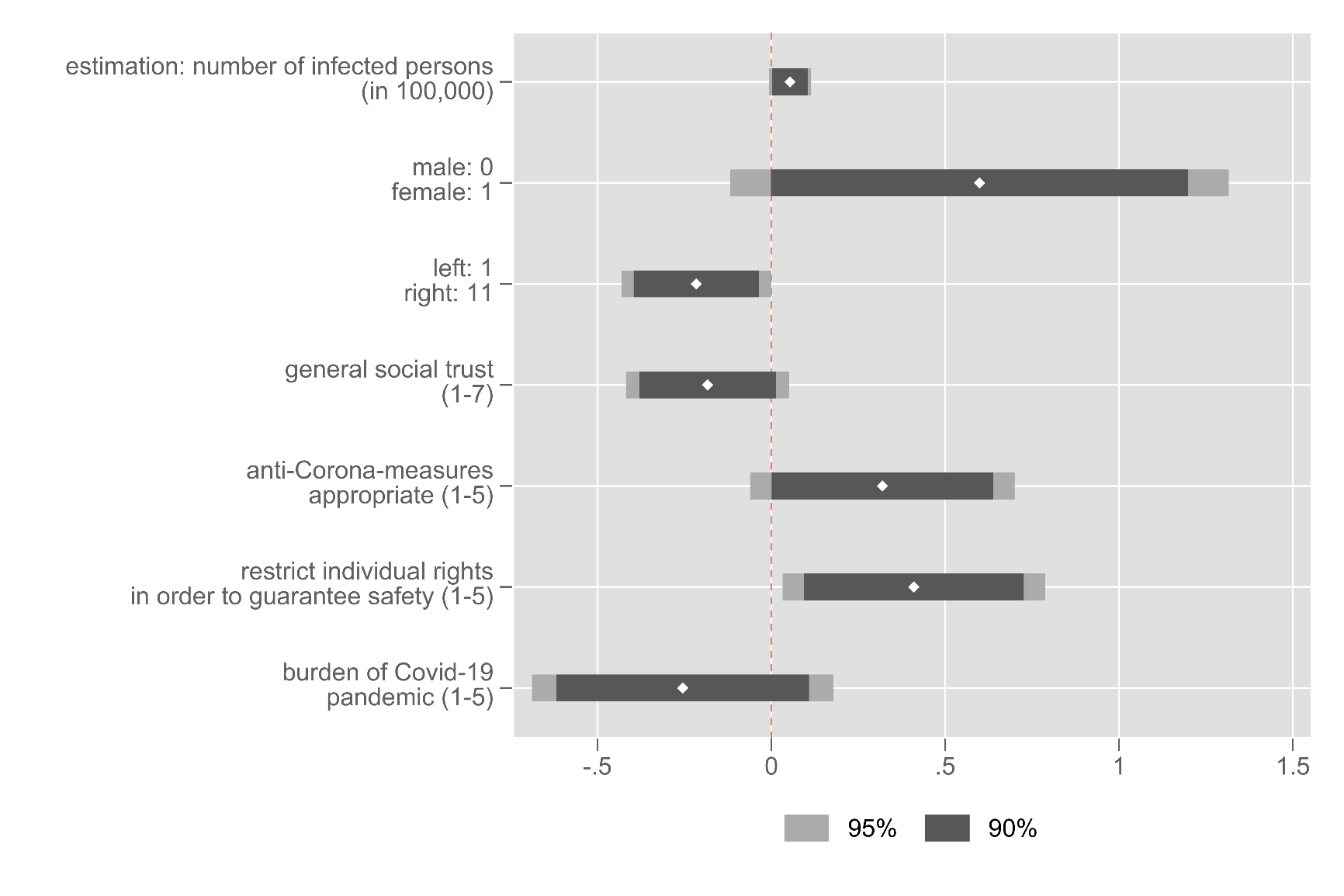


# Table A1: Descriptive overview of the variables

|  |  |  |  |  |  |  |  |
| --- | --- | --- | --- | --- | --- | --- | --- |
| **Variable** | **Exact question in the survey (in German)** | **Exact question in the survey (English translation)** | **N** | **mean** | **sd** | **min** | **max** |
| Contact restrictions | Alles in Allem, wie stehen Sie zu den folgenden Maßnahmen um die Corona-Pandemie in den Griff zu bekommen? | All in all, how do you feel about the following measures to get the Corona pandemic under control? |  |  |  |  |  |
|  | - Kontaktbeschränkungen (z.B. max. 2 Haushalte) | - Contact restrictions (e.g. max. 2 households) | 121 | 7.364 | 2.029 | 1 | 10 |
|  | - das Schließen von Restaurants | - Closing restaurants | 121 | 5.366 | 2.426 | 1 | 10 |
|  | - generelle Maskenpflicht in der Öffentlichkeit | - Wearing face masks mandatory | 121 | 8.050 | 2.264 | 1 | 10 |
|  | - Hybrid-Unterricht für Schüler*innen | - Hybrid teaching at schools | 121 | 6.992 | 2.403 | 1 | 10 |
| Treatment group | *participants were randomly assigned to the treatment/control group* | | 121 | 0.496 | 0.502 | 0 | 1 |
| Male = 0 /  female = 1 | Welches Geschlecht haben Sie? | What gender are you? | 121 | 0.545 | 0.500 | 0 | 1 |
| Left = 1 /  right = 11 | Viele verwenden die Begriffe "links" und "rechts", wenn es darum geht, unterschiedliche politische Einstellungen zu kennzeichnen. Wenn Sie an Ihre eigenen politischen Ansichten denken, wo stufen Sie sich auf dieser Links-Rechts-Skala ein? | Many use the terms "left" and "right" when referring to different political attitudes. When you think of your own political views, where do you place yourself on this left-right scale? | 121 | 3.736 | 1.632 | 1 | 11 |
| General social trust (1-7) | Ganz allgemein gesprochen: Glauben Sie, dass man den meisten Menschen vertrauen kann oder dass man im Umgang mit anderen Menschen nicht vorsichtig genug sein kann? | Speaking in general terms: Do you think that most people can be trusted or that you can't be careful enough when dealing with other people? | 121 | 4.107 | 1.493 | 1 | 7 |
| Anti-Corona-measures appropriate (1-5) | Staatliche Stellen in Deutschland haben im Zuge der Corona-Pandemie eine Vielzahl von Maßnahmen getroffen, über die man unterschiedlicher Meinung sein kann. Was denken Sie alles in allem darüber?  Ich halte die Maßnahmen für geeignet, um die gesundheitlichen Folgen der Corona-Pandemie in den Griff zu bekommen. | Government agencies in Germany have taken a variety of measures in the wake of the Corona pandemic, about which one can have different opinions. What do you think about them all in all?  I think the measures are suitable for dealing with the health consequences of the Corona pandemic. | 121 | 3.736 | 0.947 | 1 | 5 |
| Restrict individual rights in order to guarantee safety  (1-5) | Es gibt unterschiedliche Auffassungen darüber, was der Staat tun oder lassen soll. Inwieweit stimmen Sie den folgenden Aussagen zu oder nicht zu? Um die Sicherheit und das Wohlergehen der Menschen zu garantieren, sollte der Staat notfalls Rechte des Einzelnen einschränken. | There are different views on what the state should or should not do. To what extent do you agree or disagree with the following statements? In order to guarantee people's safety and well-being, the state should restrict individual rights if necessary. | 121 | 3.669 | 0.952 | 1 | 5 |
| Burden of Covid-19 pandemic (1-5) | Durch die Corona-Pandemie fühlen sich einige Menschen belastet. Wie sieht das bei Ihnen persönlich aus? Alles in allem, wie sehr fühlen Sie sich in Ihrem Leben durch die Verbreitung des Corona-Virus und die aktuellen Maßnahmen zur Bekämpfung des Virus belastet? | Some people feel burdened by the Corona pandemic. How does that look for you personally? All in all, how burdened do you feel in your life by the spread of the Corona virus and the current measures to combat it? | 121 | 3.223 | 0.821 | 1 | 5 |
| I Have been tested positive on Covid-19 | Ich war bzw. bin positiv auf das Corona-Virus getestet worden. | I was or have been tested positive for the Corona virus. | 121 | 0.025 | 0.156 | 0 | 1 |
| I Have been in quarantine | Ich war bzw. bin aufgrund eines Corona-Verdachts in Quarantäne. | I was or am in quarantine due to a suspected corona infection. | 121 | 0.306 | 0.463 | 0 | 1 |

# Table A2: Regression models – contact restrictions

|  | M1  OLS  main model | M2  OLS  robust se | M3  OLS ordinal independent variables treated as categorical | M4  Ordinal logit | M5  OLS + further controls  (own affectedness) |
| --- | --- | --- | --- | --- | --- |
|  |  |  |  |  |  |
| treatment group | 0.856^**^ | 0.856^**^ | 0.959^***^ | 1.066^***^ | 0.830^**^ |
|  | (2.43) | (2.31) | (2.64) | (3.05) | (2.23) |
|  |  |  |  |  |  |
| male = 0 / female = 1 | 0.555 | 0.555 | 0.729^**^ | 0.621^*^ | 0.514 |
|  | (1.55) | (1.49) | (2.04) | (1.80) | (1.41) |
|  |  |  |  |  |  |
| left = 1 / right = 11 | -0.249^**^ | -0.249^*^ | -0.228^*^ | -0.235^**^ | -0.257^**^ |
|  | (-2.30) | (-1.95) | (-1.86) | (-2.23) | (-2.33) |
|  |  |  |  |  |  |
| general social trust (1-7) | -0.187 | -0.187^*^ |  | -0.207^*^ | -0.191 |
|  | (-1.61) | (-1.81) |  | (-1.87) | (-1.54) |
|  |  |  |  |  |  |
| anti-Corona-measures appropriate (1-5) | 0.316^*^ | 0.316 |  | 0.259 |  |
|  | (1.67) | (1.57) |  | (1.42) |  |
|  |  |  |  |  |  |
| restrict individual rights in order to guarantee safety (1-5) | 0.428^**^ | 0.428^**^ |  | 0.400^**^ |  |
|  | (2.30) | (2.27) |  | (2.18) |  |
|  |  |  |  |  |  |
| burden of Covid-19 pandemic (1-5) | -0.298 | -0.298 |  | -0.372^*^ |  |
|  | (-1.37) | (-1.29) |  | (-1.78) |  |
|  |  |  |  |  |  |
| general social trust – 1  (you can never be too careful) |  |  | 0 |  |  |
|  |  |  | (.) |  |  |
|  |  |  |  |  |  |
| General social trust – 2 |  |  | 0.296 |  |  |
|  |  |  | (0.30) |  |  |
|  |  |  |  |  |  |
| General social trust – 3 |  |  | -1.557 |  |  |
|  |  |  | (-1.64) |  |  |
|  |  |  |  |  |  |
| General social trust – 4 |  |  | -1.077 |  |  |
|  |  |  | (-1.18) |  |  |
|  |  |  |  |  |  |
| General social trust – 5 |  |  | -0.902 |  |  |
|  |  |  | (-0.99) |  |  |
|  |  |  |  |  |  |
| General social trust – 6 |  |  | -0.160 |  |  |
|  |  |  | (-0.16) |  |  |
|  |  |  |  |  |  |
| General social trust – 7  (most people can be trusted) |  |  | -2.407^**^ |  |  |
|  |  |  | (-2.10) |  |  |
|  |  |  |  |  |  |
| anti-Corona-measures appropriate – 1  (strongly oppose) |  |  | 0 |  |  |
|  |  |  | (.) |  |  |
|  |  |  |  |  |  |
| anti-Corona-measures appropriate – 2 |  |  | -0.694 |  |  |
|  |  |  | (-0.49) |  |  |
|  |  |  |  |  |  |
| anti-Corona-measures appropriate – 3 |  |  | -1.462 |  |  |
|  |  |  | (-1.02) |  |  |
|  |  |  |  |  |  |
| anti-Corona-measures appropriate – 4 |  |  | -0.168 |  |  |
|  |  |  | (-0.12) |  |  |
|  |  |  |  |  |  |
| anti-Corona-measures appropriate – 5 (strongly support) |  |  | 0.663 |  |  |
|  |  |  | (0.47) |  |  |
|  |  |  |  |  |  |
| restrict individual rights in order to guarantee safety – 1  (strongly oppose) |  |  | 0 |  |  |
|  |  |  | (.) |  |  |
|  |  |  |  |  |  |
| restrict individual rights in order to guarantee safety – 2 |  |  | -0.178 |  |  |
|  |  |  | (-0.09) |  |  |
|  |  |  |  |  |  |
| restrict individual rights in order to guarantee safety – 3 |  |  | -0.416 |  |  |
|  |  |  | (-0.21) |  |  |
|  |  |  |  |  |  |
| restrict individual rights in order to guarantee safety – 4 |  |  | -0.197 |  |  |
|  |  |  | (-0.10) |  |  |
|  |  |  |  |  |  |
| restrict individual rights in order to guarantee safety – 5 (strongly support) |  |  | 0.821 |  |  |
|  |  |  | (0.42) |  |  |
|  |  |  |  |  |  |
| burden of Covid-19 pandemic – 1 (I feel no burden at all) |  |  | 0 |  |  |
|  |  |  | (.) |  |  |
|  |  |  |  |  |  |
| burden of Covid-19 pandemic – 2 |  |  | 0.731 |  |  |
|  |  |  | (0.23) |  |  |
|  |  |  |  |  |  |
| burden of Covid-19 pandemic – 3 |  |  | 0.870 |  |  |
|  |  |  | (0.27) |  |  |
|  |  |  |  |  |  |
| burden of Covid-19 pandemic – 4 |  |  | 0.351 |  |  |
|  |  |  | (0.11) |  |  |
|  |  |  |  |  |  |
| burden of Covid-19 pandemic – 5 (I feel a very big burden) |  |  | 0.127 |  |  |
|  |  |  | (0.04) |  |  |
|  |  |  |  |  |  |
| I have been in quarantine |  |  |  |  | -0.128 |
|  |  |  |  |  | (-0.32) |
|  |  |  |  |  |  |
| I have been tested positive on Covid-19 |  |  |  |  | 0.269 |
|  |  |  |  |  | (0.23) |
|  |  |  |  |  |  |
| Constant | 6.551^***^ | 6.551^***^ | 7.916^***^ |  | 8.447^***^ |
|  | (4.91) | (4.27) | (3.04) |  | (11.82) |
| Observations | 121 | 121 | 121 | 121 | 121 |
| Adjusted *R*^2^ | 0.15 | 0.15 | 0.22 |  | 0.07 |

*b-coefficients, t* statistics in parentheses; ^*^ *p* < 0.10, ^**^ *p* < 0.05, ^***^ *p* < 0.01

# Table A3: Quotes by German politicians about the coronavirus pandemic including the term “exponential”

| **Date** | **Name** | **Position** | **Bundesland** | **Quote in German** | **Quote English translation** | **Source** |
| --- | --- | --- | --- | --- | --- | --- |
| 13.03.2020 | Christine Streichert-Clivot | Bildungsministerin | Rheinland-Pfalz | Es geht darum, das extreme, exponentielle Ansteigen der Fallzahlen zu durchbrechen, insbesondere um besonders verletzliche Bevölkerungsgruppen zu schützen | The aim is to break the extreme, exponential increase in the number of cases, particularly in order to protect vulnerable populations | https://www.sueddeutsche.de/gesundheit/krankheiten-mainz-regierung-beraet-ueber-schliessung-von-schulen-und-kitas-dpa.urn-newsml-dpa-com-20090101-200313-99-314827 |
| 22.03.2020 | Sabine Bätzing-Lichtenthäler | Gesundheitsministerin | Rheinland-Pfalz | Wir haben die Hoffnung, dass diese exponentielle Steigerung in der nächsten Woche nicht mehr so stark sein wird, dass man es schafft, die Kurve abzuflachen, nach hinten zu schieben | We have hope that this exponential increase will not be so strong in the next week that you manage to flatten the curve, push it backwards | https://www.sueddeutsche.de/gesundheit/gesundheit-mainz-corona-hotspots-an-der-unteren-mosel-und-in-rheinhessen-dpa.urn-newsml-dpa-com-20090101-200321-99-417154 |
| 27.03.2020 | Tarek Al-Wazir | Wirtschaftsminister | Hessen | Indirekt: Wenn die exponentielle Ausbreitung des Coronavirus gebrochen sei, müsse man sich natürlich Gedanken machen, wie das Land schrittweise zur Normalität zurückfinden könne, ohne eine neue Infektionswelle zu erleben. | Indirectly: Once the exponential spread of the coronavirus is broken, it is natural to think about how the country can gradually return to normality without experiencing a new wave of infection. | https://www.sueddeutsche.de/gesundheit/gesundheit-wiesbaden-wirtschaftsminister-hessen-haerter-als-andere-getroffen-dpa.urn-newsml-dpa-com-20090101-200327-99-492032 |
| 30.03.2020 | Markus Söder | Ministerpräsident | Bayern | Immer noch sei die Entwicklung in Bayern exponentiell. Auch Jüngere könne es treffen. | The development in Bavaria is still exponential. Younger people could also be affected. | https://www.sueddeutsche.de/bayern/coronavirus-bayern-ausgangsbeschraenkung-dauer-1.4862126 |
| 03.04.2020 | Markus Söder | Ministerpräsident | Bayern | Wir befinden uns immer noch in einer Phase des exponentiellen Wachstums | We are still in a phase of exponential growth | https://www.sueddeutsche.de/bayern/coronavirus-bayern-rueckblick-april-1.4873340 |
| 03.04.2020 | Manne Lucha | Gesundheitsminister | Baden-Württemberg | Indirekt: Die Neuerkrankungsraten seien in den vergangenen Tagen aber nicht mehr exponentiell gestiegen, sondern auf ähnlichem Niveau stabil geblieben, sagte Lucha. "Wir rüsten uns, dass wir an den Ostertagen maximal einsatzfähig sind." | Indirectly: Lucha said new illness rates have stopped increasing exponentially in recent days and have remained stable at similar levels. "We're gearing up to be at maximum readiness for Easter." | https://www.sueddeutsche.de/gesundheit/gesundheit-stuttgart-aerzte-pfleger-und-das-virus-wenn-sich-helfer-infizieren-dpa.urn-newsml-dpa-com-20090101-200403-99-577680 |
| 06.04.2020 | Tarek Al-Wazir | Wirtschaftsminister | Hessen | Wir müssen das exponentielle Wachstum der Ausbreitung der Infektionen brechen." Ansonsten käme das Land in eine Situation, in der das Gesundheitswesen überlastet wäre. | We need to break the exponential growth of the spread of infections." Otherwise, the country would get into a situation where the healthcare system would be overburdened. | https://www.sueddeutsche.de/gesundheit/gesundheit-wiesbaden-al-wazir-bip-koennte-um-bis-zu-zehn-prozent-einbrechen-dpa.urn-newsml-dpa-com-20090101-200406-99-601962 |
| 19.04.2020 | Helge Braun | Kanzleramtschef | Bund | Indirekt: Die zu Beginn der Ausbreitung des Coronavirus in Deutschland von der Kanzlerin genannte Verdopplungszeit der Infektionszahlen sei ein wichtiger Indikator, wenn die Zahlen akut exponentiell steigen würden, wie Mitte März, sagte Braun. "Das ist zurzeit zum Glück nicht mehr der Fall - und doch sollten wir sie im Auge behalten." | Indirectly: the doubling time of infection numbers cited by the chancellor at the beginning of the spread of coronavirus in Germany is an important indicator when numbers are acutely exponential, as they were in mid-March, Braun said. "Fortunately, that is no longer the case at the moment - but we should still keep an eye on them." | https://www.sueddeutsche.de/gesundheit/gesundheit-kanzleramtschef-herdenimmunitaet-untauglich-gegen-corona-dpa.urn-newsml-dpa-com-20090101-200419-99-750268 |
| 23.04.2020 | Kai Klose | Sozialminister | Hessen | Umgekehrt müssen wir auch mutig genug sein, bei den Lockerungen einen Schritt zurückzugehen, wenn die Anzahl der Fälle wieder exponentiell wachsen sollte. Das gehört zur Wahrheit dazu | Conversely, we must also be brave enough to take a step back in relaxations if the number of cases should grow exponentially again. That is part of the truth | https://www.sueddeutsche.de/gesundheit/krankheiten-wiesbaden-kai-klose-zu-corona-lockerungen-diszipliniert-bleiben-dpa.urn-newsml-dpa-com-20090101-200423-99-811450 |
| 29.04.2020 | Ursula Nonnemacher | Gesundheitsministerin | Brandenburg | Indirekt: Sie warne aber, "wenn wir auf einen Schlag wieder zuviel freigeben, dass dann die Gefahr besteht, dass wir wieder in ein exponentielles Wachstum kommen." | Indirect: Though, she warns, "if we release too much again in one fell swoop, that there's then a danger that we'll get back into exponential growth." | https://www.sueddeutsche.de/gesundheit/gesundheit-potsdam-brandenburg-will-corona-reserve-in-krankenhaeusern-senken-dpa.urn-newsml-dpa-com-20090101-200429-99-876593 |
| 30.04.2020 | Dietmar Woidke | Ministerpräsident | Brandenburg | Wir haben mittlerweile eine stabile Situation. Es ist nicht zu einer exponentiellen Verbreitung des Virus gekommen und damit auch nicht zu einer Überforderung des Gesundheitssystems | We now have a stable situation. There has been no exponential spread of the virus and thus no overtaxing of the health care system. | https://www.sueddeutsche.de/gesundheit/krankheiten-potsdam-woidke-fuer-einheitliches-vorgehen-der-laender-in-corona-krise-dpa.urn-newsml-dpa-com-20090101-200430-99-889685 |
| 01.05.2020 | Angela Merkel | Bundeskanzlerin | Bund | Indirekt: Am Donnerstag verteidigte Merkel dennoch das Herausstellen der Verdoppelungszahl: Damit habe man zeigen können, was "exponentielles Wachstum" bedeute. Für den Anfang sei das "unheimlich einprägsam" gewesen. | Indirectly: On Thursday, Merkel nevertheless defended the highlighting of the doubling figure, saying that it had made it possible to show what "exponential growth" meant. For a start, that was "incredibly memorable. | https://www.sueddeutsche.de/politik/corona-krise-in-deutschland-was-fuer-ein-zustand-1.4893967 |
| 09.05.2020 | Stephan Weil | Ministerpräsident | Niedersachsen | Wir haben die exponentielle Kurve bei den Infektionen erfolgreich bekämpft, aber stecken jetzt in einer anderen exponentiellen Kurve. Das sind die wachsenden Schäden in Gesellschaft und Wirtschaft. Um die müssen wir uns dringend kümmern. | We have successfully fought the exponential curve in infections, but are now stuck in another exponential curve. That is the growing damage to society and the economy. We urgently need to take care of these. | https://www.sueddeutsche.de/gesundheit/gesundheit-hannover-weil-auf-sicht-fahren-stoesst-auf-immer-weniger-verstaendnis-dpa.urn-newsml-dpa-com-20090101-200509-99-996249 |
| 04.07.2020 | Angela Merkel | Bundeskanzlerin | Bund | Indirekt: Im Gegensatz zu Söder könne sie sich eine Verlängerung "jetzt nicht vorstellen, weil ich auch daran arbeiten werde, ... , dass es nicht wieder zu einem Ausbruch exponentieller Art von Covid-19-Fällen kommt." | Indirectly: Unlike Söder, she could not imagine an extension "now, because I will also work to ... , that there is not another outbreak of an exponential nature of Covid 19 cases." | https://www.sueddeutsche.de/politik/bundesregierung-130-milliarden-gegen-die-krise-viel-lob-fuer-schwarz-rot-dpa.urn-newsml-dpa-com-20090101-200604-99-299229 |
| 28.07.2020 | Winfried Kretschmann | Ministerpräsident | Baden-Württemberg | Indirekt: Man müsse damit rechnen, dass es wieder mehr Infektionsketten geben und das Virus auch von Reiserückkehrern stärker importiert werde. Entscheidend sei allerdings, ob es zu einer exponentiellen Kurve der Infektionen komme. | Indirectly: It must be expected that there will be more chains of infection again and that the virus will also be imported more strongly by people returning from travel. The decisive factor, however, is whether there will be an exponential curve of infections. | https://www.sueddeutsche.de/gesundheit/gesundheit-stuttgart-corona-verstoesse-kretschmann-droht-mit-haerteren-sanktionen-dpa.urn-newsml-dpa-com-20090101-200728-99-952423 |
| 24.08.2020 | Markus Söder | Ministerpräsident | Bayern | Indirekt: "Wir stehen an einer ganz wichtigen Weggabelung", betonte Söder. "Corona ist wieder voll da". Wie im Frühjahr sei es kurz davor, dass es in Deutschland wieder eine exponentielle Entwicklung bei den Fallzahlen gebe. Ziel der Politik müsse es aber sein, vor die Welle zu kommen. | Indirectly: "We are at a very important crossroads," Söder emphasized. "Corona is back in full force". As in the spring, he said, it was on the verge of another exponential development in the number of cases in Germany. The goal of politics, however, must be to get ahead of the wave, he said. | https://www.sueddeutsche.de/gesundheit/gesundheit-muenchen-soeder-fordert-bundesweite-standards-im-kampf-gegen-corona-dpa.urn-newsml-dpa-com-20090101-200824-99-286448 |
| 01.09.2020 | Florian Herrmann | Staatsminister | Bayern | Indirekt: Die Staatsregierung versuche daher, das Virus Sars-CoV-2 so weit wie möglich im Griff zu behalten, "um nicht dann wieder einer exponentiellen Entwicklung ausgeliefert zu sein, die dann unweigerlich wieder sicher dazu führen wird, dass das Gesundheitssystem unter Druck kommt, dass auch dann die schweren Erkrankungen schlimmer werden". | Indirect: The state government is therefore trying to keep the Sars-CoV-2 virus under control as much as possible, "so as not to then be at the mercy of an exponential development again, which will then inevitably lead to the health system coming under pressure again for sure, that the serious illnesses will then also become worse." | https://www.sueddeutsche.de/gesundheit/gesundheit-muenchen-staatsminister-herrmann-verteidigt-corona-massnahmen-dpa.urn-newsml-dpa-com-20090101-200901-99-389032 |
| 06.09.2020 | Markus Söder | Ministerpräsident | Bayern | Aber um uns herum gibt es wieder exponentielle Entwicklungen. In Spanien, Frankreich, Tschechien oder Italien sind es die höchsten Zahlen seit Mai. | But around us there are exponential developments again. In Spain, France, the Czech Republic or Italy, the numbers are the highest since May. | https://www.sueddeutsche.de/gesundheit/gesundheit-wuerzburg-warten-auf-das-corona-testergebnis-warum-es-dauern-kann-dpa.urn-newsml-dpa-com-20090101-200905-99-443512 |
| 11.09.2020 | Markus Söder | Ministerpräsident | Bayern | Indirekt: Entscheidend sei, dass eine exponentielle Kurve auf alle Fälle vermieden werden müsse, um einen weiteren Lockdown zu vermeiden. | Indirectly: The decisive factor is that an exponential curve must be avoided at all costs in order to prevent a further lockdown. | https://www.sueddeutsche.de/politik/regierung-nuernberg-soeder-zu-viel-unvernunft-im-umgang-mit-corona-dpa.urn-newsml-dpa-com-20090101-200911-99-521964 |
| 30.09.2020 | Angela Merkel | Bundeskanzlerin | Bund | Indirekt: Sie habe das Infektionsgeschehen der vergangenen drei Monate genommen, als sich die Zahlen jeweils von rund 300 auf 600, dann auf 1200 und schließlich auf 2400 verdoppelt hatten. Rechne man dasselbe exponentielle Wachstum für weitere drei Monate hoch, komme man über 4800 und 9600 schließlich auf 19200 bis Weihnachten. | Indirectly: She had taken the infection events of the past three months, when the numbers had doubled in each case from about 300 to 600, then to 1200 and finally to 2400. If one extrapolates the same exponential growth for another three months, one arrives via 4800 and 9600 finally at 19200 by Christmas. | https://www.sueddeutsche.de/politik/angela-merkel-corona-bundestag-1.5050943 |
| 10.10.2020 | Markus Söder | Ministerpräsident | Bayern | Leider sind wir auf dem Weg zu exponenziellem Wachstum, gerade in den Großstädten ist die Herausforderung sehr, sehr groß. | Unfortunately, we are on the path to exponential growth, especially in the big cities, the challenge is very, very great. | https://www.sueddeutsche.de/gesundheit/krankheiten-muenchen-soeder-zu-corona-auf-dem-weg-zu-exponentiellem-wachstum-dpa.urn-newsml-dpa-com-20090101-201010-99-896697 |
| 15.10.2020 | Carola Reimann | Gesundheitsministerin | Niedersachsen | Es steht zu befürchten, dass wir erneut in eine Phase des exponentiellen Wachstums eintreten, wenn wir jetzt nicht konsequent gegensteuern. | It is to be feared that we will once again enter a phase of exponential growth if we do not take consistent countermeasures now. | https://www.sueddeutsche.de/gesundheit/gesundheit-hannover-reimann-nennt-corona-entwicklung-besorgniserregend-dpa.urn-newsml-dpa-com-20090101-201015-99-956024 |
| 15.10.2020 | Daniel Günther | Ministerpräsident | Schleswig Holstein | Wir stehen in Deutschland am Anfang eines ungebremsten exponenteillen Wachstums der Infektionszahlen und müssen dringend jetzt eine Haltelinie definieren. | In Germany, we are at the beginning of an unchecked exponential growth in the number of infections and urgently need to define a stop line now. | https://www.sueddeutsche.de/wirtschaft/gastgewerbe-kiel-guenther-schleswig-holstein-haelt-an-beherbergungsregeln-fest-dpa.urn-newsml-dpa-com-20090101-201015-99-954760 |
| 16.10.2020 | Felix Schwenke | Oberbürgermeister Offenbach am Main | Hessen | Indirekt: Oberbürgermeister Felix Schwenke (SPD) sprach vom Beginn eines neuen exponentiellen Wachstums. Er rief Bürger auf, die Zahl ihrer Kontakte zu beschränken. Auch Feiern müssten weniger werden. | Indirectly: Mayor Felix Schwenke (SPD) spoke of the beginning of a new exponential growth. He called on citizens to limit the number of contacts they have. Celebrations would also have to become fewer. | https://www.sueddeutsche.de/gesundheit/gesundheit-offenbach-am-main-offenbach-schraenkt-kontakte-ein-und-weitet-maskenpflicht-aus-dpa.urn-newsml-dpa-com-20090101-201016-99-970740#https://www.sueddeutsche.de/gesundheit/gesundheit-offenbach-am-main-offenbach-schraenkt-kontakte-ein-und-weitet-maskenpflicht-aus-dpa.urn-newsml-dpa-com-20090101-201016-99-970740 |
| 19.10.2020 | Dietmar Woidke | Ministerpräsident | Brandenburg | Indirekt: Deutschland stehe am Beginn eines exponentiellen Wachstums, betonte Woidke am Montag im ARD-"Morgenmagazin". | Indirectly: Germany is at the beginning of an exponential growth, Woidke emphasized on Monday in the ARD "Morgenmagazin". | https://www.sueddeutsche.de/gesundheit/gesundheit-potsdam-brandenburg-vor-weiteren-corona-beschraenkungen-dpa.urn-newsml-dpa-com-20090101-201019-99-996824 |
| 19.10.2020 | Michael Kretschmer | Ministerpräsident | Sachsen | Wir sind in der exponentiellen Phase | We are in the exponential phase | https://www.sueddeutsche.de/gesundheit/gesundheit-lauterbach-zaehlt-auf-das-verhalten-der-buerger-dpa.urn-newsml-dpa-com-20090101-201018-99-984024 |
| 21.10.2020 | Markus Söder | Ministerpräsident | Bayern | Indirekt: Noch habe man "kein absolut exponentielles Wachstum", sagte er mit Blick auf die Infektionszahlen in Bayern. | Indirectly: Still one has "no absolutely exponential growth", he said with view of the infection numbers in Bavaria. | https://www.sueddeutsche.de/bayern/bayern-coronavirus-soeder-regierungserklaerung-1.5087064 |
| 29.10.2020 | Angela Merkel | Bundeskanzlerin | Bund | Indirekt: Entwickelte Hygienekonzepte würden wieder gebraucht werden, könnten "im gegenwärtigen exponentiellen Infektionsgeschehen ihre Kraft aber nicht entfalten." | Indirect: Developed hygiene concepts would be needed again, but could not "unleash their power in the current exponential infection event." | https://www.sueddeutsche.de/politik/corona-bundestag-regierungserklaerung-merkel-1.5097789 |
| 29.10.2020 | Karl Lauterbach | SPD-Gesundheitsexperte | Bund | Indirekt: Mit dem "Wellenbrecher Shutdown" werde man die zweite Welle der Pandemie brechen und aus dem exponentiellen Wachstum herauskommen. | Indirectly, he said, the "breakwater shutdown" would break the second wave of the pandemic and bring us out of exponential growth. | https://www.sueddeutsche.de/politik/corona-massnahmen-november-reaktionen-1.5097790 |
| 29.10.2020 | Sabine Bätzing-Lichtenthäler | Gesundheitsministerin | Rheinland-Pfalz | Wir sind am Beginn eines exponentiellen Wachstums (...). Diese Dynamik bei den Infektionszahlen führt dazu, dass wir als Politik eingreifen müssen. | We are at the beginning of an exponential growth (...). This dynamic in the number of infections means that we as policymakers have to intervene. | https://www.sueddeutsche.de/gesundheit/gesundheit-mainz-zahl-der-covid-patienten-in-kliniken-fast-versechsfacht-dpa.urn-newsml-dpa-com-20090101-201029-99-130803 |
| 29.10.2020 | Malu Dreyer | Ministerpräsidentin | Rheinland-Pfalz | Indirekt: Es sei ein gutes Signal, dass sich Bund und Länder am Beginn des exponentiellen Wachstums des Coronavirus auf einheitliche Regeln verständigt hätten,"und wir wieder geschlossen miteinander vorgehen", sagte Dreyer. | Indirectly: Dreyer said it was a good signal that the federal and state governments had agreed on uniform rules at the beginning of the exponential growth of the coronavirus, "and that we are once again acting in concert with each other. | https://www.sueddeutsche.de/gesundheit/gesundheit-mainz-corona-kabinett-beraet-ueber-massnahmen-dpa.urn-newsml-dpa-com-20090101-201029-99-124361 |
| 30.10.2020 | Dirk Toepffer | CDU-Fraktionschef | Niedersachsen | Indirekt: Nur wenige Verweigerer könnten enormen Einfluss auf das exponentielle Infektionsgeschehen nehmen | Indirect: Just a few holdouts could have enormous impact on exponential infection events | https://www.sueddeutsche.de/politik/landtag-hannover-landtag-debattiert-ueber-bevorstehenden-corona-teil-lockdown-dpa.urn-newsml-dpa-com-20090101-201029-99-134538 |
| 02.11.2020 | Angela Merkel | Bundeskanzlerin | Bund | Das ist exponentielles Wachstum, das uns mit zunehmender Geschwindigkeit auf eine akute Notlage in unseren Krankenhäusern zulaufen lässt. | This is exponential growth that has us running at an increasing rate toward an acute emergency in our hospitals. | https://www.sueddeutsche.de/gesundheit/gesundheit-deutschland-im-teil-lockdown-dpa.urn-newsml-dpa-com-20090101-201102-99-171104 |
| 12.11.2020 | Carola Reimann | Gesundheitsministerin | Niedersachsen | Wenn wir uns alle weiterhin an die Regeln halten und diszipliniert verhalten, besteht aber die Hoffnung, dass wir den vorläufigen Höhepunkt bei den Neuinfektionen bald hinter uns haben und das exponentielle Wachstum bremsen können. | However, if we all continue to follow the rules and behave in a disciplined manner, there is hope that we will soon be past the temporary peak in new infections and be able to slow down the exponential growth. | https://www.sueddeutsche.de/gesundheit/gesundheit-hannover-zahl-neuer-corona-infektionen-steigt-leicht-dpa.urn-newsml-dpa-com-20090101-201111-99-293887 |
| 13.11.2020 | Karl Lauterbach | SPD-Gesundheitsexperte | Bund | Wir sind aus dem exponentiellen Wachstum heraus. Das ist ein großer Erfolg. | We are out of exponential growth. That is a great success. | https://www.sueddeutsche.de/politik/bundesregierung-bund-will-corona-einschraenkungen-nicht-lockern-dpa.urn-newsml-dpa-com-20090101-201113-99-316985 |
| 16.11.2020 | Angela Merkel | Bundeskanzlerin | Bund | Indirekt: Merkel sagte nach den Beratungen am Montag, man sei durch das Schließen bestimmter Einrichtungen im Freizeitbereich aus dem exponentiellen Wachstum herausgekommen und man müsse nun darüber sprechen, ob man das zum 1. Dezember alles wieder öffnen könne oder nicht. | Indirectly: Merkel said after Monday's deliberations, you're out of exponential growth by closing certain facilities in the recreation sector and you now have to talk about whether or not you can reopen all of that by Dec. 1. | https://www.sueddeutsche.de/politik/bundesregierung-wie-geht-s-weiter-im-corona-winter-dpa.urn-newsml-dpa-com-20090101-201116-99-355688 |
| 16.11.2020 | Angela Merkel | Bundeskanzlerin | Bund | Indirekt: Das exponentielle Wachstum sei gestoppt, eine Trendumkehr aber noch nicht erreicht | Indirect: Exponential growth has stopped, but a trend reversal has not yet been achieved | https://www.sueddeutsche.de/politik/corona-merkel-laender-1.5117416 |
| 16.11.2020 | Winfried Kretschmann | Ministerpräsident | Baden-Württemberg | Steigen die Zahlen weiter exponentiell an, wird das zur Überlastung der Krankenhäuser führen. | If the numbers continue to rise exponentially, it will lead to hospital overcrowding. | https://www.sueddeutsche.de/politik/coronavirus-news-rki-beschraenkungen-1.5109218 |
| 16.11.2020 | Kathrin Schneider | Staatskanzleichefin | Brandenburg | Durch die Einschränkungen Anfang November konnte das exponentielle Wachstum der Infektionen gebremst werden | Restrictions in early November slowed the exponential growth of infections | https://www.sueddeutsche.de/gesundheit/gesundheit-potsdam-woidke-keine-zeit-fuer-lockerungen-dpa.urn-newsml-dpa-com-20090101-201116-99-355428 |
| 17.11.2020 | Angela Merkel | Bundeskanzlerin | Bund | Indirekt: Deutschland müsse in Zukunft schneller agieren, wenn sich ein exponentielles Wachstum abzeichne - auch wenn man noch nichts auf den Intensivstationen sehe. | Indirectly: he said, Germany will have to act more quickly in the future if exponential growth is on the horizon - even if nothing is seen yet in the intensive care units. | https://www.sueddeutsche.de/politik/corona-pandemie-ich-bin-da-manchmal-ungeduldig-1.5118400 |
| 18.11.2020 | Armin Laschet | Ministerpräsident | Nordrhein-Westfalen | Das exponentielle Wachstum ist gebrochen. | The exponential growth is broken. | https://www.sueddeutsche.de/wirtschaft/armin-laschet-nrw-corona-1.5119212 |
| 18.11.2020 | Jens Spahn | Gesundheitsminister | Bund | Indirekt: "Wir haben Tritt gefasst." Das exponentielle Wachstum der Neuinfektionen sei gestoppt, "aber wir sind noch nicht über den Berg". | Indirectly: "We have found our footing." The exponential growth of new infections has stopped, "but we're not out of the woods yet." | https://www.sueddeutsche.de/politik/demonstrationen-berlin-reform-des-infektionsschutzgesetzes-beschlossen-proteste-dpa.urn-newsml-dpa-com-20090101-201118-99-379465 |
| 18.11.2020 | Jens Spahn | Gesundheitsminister | Bund | Indirekt: Die körperliche Unversehrtheit stehe auch im Grundgesetz, sagte Spahn, und der Schutz der Gesundheit stehe nicht absolut über allem. Aber die Regierung habe entschieden, ihr ein relativ starkes Gewicht beizumessen. Auch um eine Überforderung des Gesundheitssystems zu verhindern, zu dem es bei einem exponentiellen Wachstum der Infektionszahlen kommen würde. | Indirectly: Physical integrity is also in the Basic Law, said Spahn, and the protection of health is not absolutely above everything. But the government has decided to give it a relatively strong weight, he said. This is also to prevent the health care system from being overburdened, which would occur if the number of infections were to grow exponentially. | https://www.sueddeutsche.de/politik/berlin-demo-infektionsschutzgesetz-bundestag-1.5119121 |
| 22.11.2020 | Michael Müller | Regierender Bürgermeister | Berlin | Wir sind uns einig, dass schon viel erreicht wurde, aber nicht genug. Zwar konnte das exponentielle Wachstum ausgebremst werden, aber die infektionszahlen sind weiterhin zu hoch. | We agree that much has been achieved, but not enough. The exponential growth has been slowed, but the number of infections is still too high. | https://www.sueddeutsche.de/politik/bundeslaender-berlin-mueller-corona-massnahmen-koennen-noch-nicht-aufgehoben-werden-dpa.urn-newsml-dpa-com-20090101-201122-99-422787 |
| 23.11.2020 | Jens Spahn | Gesundheitsminister | Bund | Das exponentielle Wachstum ist gebrochen. Wir sind uns einig, dass das nicht reicht. Aber es ist gelungen - einmal mehr. | Exponential growth is broken. We agree that this is not enough. But it has succeeded - once again. | https://www.sueddeutsche.de/politik/corona-news-1.5116448 |
| 24.11.2020 | Marcel Schweitzer | Senatssprecher | Hamburg | Alle Maßnahmen im Bereich der Kontaktbeschränkung haben dazu beigetragen, dass wir das exponentielle Wachstum gebremst und die Welle gebrochen haben. | All the measures taken in the area of contact restriction have helped us to slow down the exponential growth and break the wave. | https://www.sueddeutsche.de/politik/senat-hamburg-hamburger-senat-positive-zwischenbilanz-des-teil-lockdowns-dpa.urn-newsml-dpa-com-20090101-201124-99-445365 |
| 25.11.2020 | Angela Merkel | Bundeskanzlerin | Bund | Indirekt: Bundeskanzlerin Angela Merkel (CDU) sprach nach der mehrstündigen Videokonferenz mit den Ministerpräsidenten von einem "Teilerfolg", weil das exponentielle Wachstum der Infektionen gebrochen sei. | Indirectly: After the video conference with the prime ministers, which lasted several hours, German Chancellor Angela Merkel (CDU) spoke of a "partial success" because the exponential growth of infections had been broken. | https://www.sueddeutsche.de/politik/pandemie-corona-regeln-werden-verschaerft-1.5127842 |
| 25.11.2020 | Stephan Weil | Ministerpräsident | Niedersachsen | Wir befinden uns bei den täglichen Neuinfektionen nach wie vor auf einem hohen Plateau, die exponentielle Steigerung der Infektionszahlen aber konnten wir gemeinsam abschwächen. | We are still at a high plateau in terms of new daily infections, but together we have been able to mitigate the exponential increase in the number of infections. | https://www.sueddeutsche.de/politik/bundesregierung-hannover-weil-corona-beschraenkungen-bis-anfang-januar-erforderlich-dpa.urn-newsml-dpa-com-20090101-201125-99-465224 |
| 26.11.2020 | Angela Merkel | Bundeskanzlerin | Bund | Indirekt: Das "dramatische, exponentielle Wachstum" der Infektionszahlen sei mit den Ende Oktober beschlossenen Beschränkungen gestoppt worden. Allerdings gebe es " noch keine Trendwende". | Indirectly: The "dramatic, exponential growth" in the number of infections has been stopped with the restrictions adopted at the end of October. However, there is " no reversal of the trend yet". | https://www.sueddeutsche.de/politik/merkel-corona-regeln-massnahmen-1.5129028 |
| 26.11.2020 | Armin Laschet | Ministerpräsident | Nordrhein-Westfalen | Indirekt: Das exponentielle Wachstum der Corona-Zahlen sei gestoppt worden, sagt Laschet | Indirectly: the exponential growth of Corona numbers has been stopped, says Laschet | https://www.sueddeutsche.de/politik/landtag-duesseldorf-laschet-verschaerfte-corona-auflagen-bis-januar-dpa.urn-newsml-dpa-com-20090101-201126-99-470439 |
| 30.11.2020 | Markus Söder | Ministerpräsident | Bayern | Indirekt: Es gebe ein exponentielles Wachstum mehr nach oben. | Indirectly: there is more upward exponential growth. | https://www.sueddeutsche.de/bayern/gesundheit-muenchen-soeder-zu-corona-regeln-viele-suchen-nach-schlupfloechern-dpa.urn-newsml-dpa-com-20090101-201130-99-512285 |
| 03.12.2020 | Angela Merkel | Bundeskanzlerin | Bund | Indirekt: So gebe es "beachtliche" Rückgänge der Infektionszahlen in Bremen, Hamburg, dem Saarland und in Niedersachsen. Dagegen weise Sachsen ein exponentielles Wachstum auf, in Thüringen und in Sachsen-Anhalt stiegen die Zahlen ebenfalls noch, aber auf niedrigerem Niveau. | Indirectly: there are "considerable" decreases in the number of infections in Bremen, Hamburg, Saarland and Lower Saxony. In contrast, Saxony is showing exponential growth, and in Thuringia and Saxony-Anhalt the numbers are also still rising, but at a lower level. | https://www.sueddeutsche.de/politik/corona-merkel-massnahmen-lockdown-1.5135747 |
| 03.12.2020 | Hubert Aiwanger | Wirtschaftsminister | Bayern | Wir wollen keinen Dauer-Lockdown, die Lage ist jetzt verglichen mit dem exponentiellen Wachstum im Oktober wieder beherrschbar | We do not want a permanent lockdown, the situation is now manageable again compared to the exponential growth in October | https://www.sueddeutsche.de/bayern/regierung-passau-aiwanger-fahrplan-fuer-corona-lockerungen-ab-11-januar-dpa.urn-newsml-dpa-com-20090101-201203-99-559531 |
| 08.12.2020 | Winfried Kretschmann | Ministerpräsident | Baden-Württemberg | Es deutet sich an, dass das exponentielle Wachstum zurück sein könnte. | There are indications that exponential growth may be back. | https://www.sueddeutsche.de/gesundheit/gesundheit-stuttgart-kretschmann-dringt-auf-scharfen-corona-lockdown-dpa.urn-newsml-dpa-com-20090101-201208-99-613820 |
| 08.12.2020 | Tobias Hans | Ministerpräsident | Saarland | Indirekt: Ministerpräsident Tobias Hans (CDU) sagte nach einer Kabinettssitzung, dass das exponentielle Wachstum der Corona-Infektionszahlen zwar gestoppt worden sie. Die bisherigen Maßnahmen (...) habe aber nicht zu einem spürbaren Rückgang geführt. | Indirectly: Prime Minister Tobias Hans (CDU) said after a cabinet meeting that the exponential growth of the Corona infection numbers had been stopped. The measures taken so far (...) but had not led to a noticeable decline. | https://www.sueddeutsche.de/politik/regierung-saarland-lockert-regeln-fuer-weihnachten-danach-verschaerfung-dpa.urn-newsml-dpa-com-20090101-201208-99-611568+ |
